# Two new species of *Parahesione* (Annelida: Hesionidae) associated with ghost shrimps (Crustacea: Decapoda) and their phylogenetic relationships

**DOI:** 10.7717/peerj.16346

**Published:** 2023-11-01

**Authors:** Naoto Jimi, Hiroki Nakajima, Taigi Sato, Brett C. Gonzalez, Sau Pinn Woo, Greg W. Rouse, Temir Britayev

**Affiliations:** 1 Nagoya University, Toba, Japan; 2 Centre for Marine and Coastal Studies, Universiti Sains Malaysia, Penang, Malaysia; 3 University of the Ryukyus, Okinawa, Japan; 4 Smithsonian Institution, National Museum of Natural History, Department of Invertebrate Zoology, Washington D.C., United States; 5 Universiti Sains Malaysia, Penang, Malaysia; 6 Scripps Institution of Oceanography, California, United States; 7 AN Severtzov Institute of Ecology and Evolution, Moscow, Russia

**Keywords:** Ryukyu Islands, Papua New Guinea, Decapoda, Polychaeta, Polychaetes, Symbiosis

## Abstract

Two new species of Hesionidae, *Parahesione pulvinata* sp. nov. and *Parahesione apiculata* sp. nov. are described based on materials collected at tidal flats in Okinawa (Japan) from burrows of the ghost shrimps *Neocallichirus jousseaumei* and *Glypturus armatus*. The two new species are characterized by having eight enlarged cirri, dorsal cirrophores with dorsal foliose lobe and biramous parapodia, and by lacking median antenna. *Parahesione apiculata* sp. nov. has digitate lobes on the posterior margin of the dorsal foliose lobe (absent in *P*. *pulvinata* sp. nov.). The two new species were never found outside the ghost shrimp burrows, suggesting they are obligate symbionts. Phylogenetic analyses based on four concatenated genes suggest that the symbiotic lifestyle has evolved several times in Hesionidae.

## Introduction

The narrow burrows excavated by decapods in tidal flats are frequently occupied by different symbionts (Campos, Campos & Manriquez, 2009; Pillay & Branch, 2011). However, these secure habitats often exist under hypoxic conditions (Atkinson & Taylor, 2005), leading to the adaptation of certain symbionts (Pillay & Branch, 2011). These include polynoid and hesionid polychaetes living in burrows of callianassid ghost shrimps and upogebiid mud shrimps (Martin & Britayev, 1998).

Hesionidae includes more than 199 species (Rouse, Carvajal & Pleijel, 2018; Rouse, Pleijel & Tilic, 2022; Read & Fauchald, 2023), with about 30 being considered obligate or facultative invertebrate symbionts, mainly living in association with echinoderms, but also with burrowing sipunculids, hemichordates and polychaetes, among others (Martin & Britayev, 1998, 2018; Martin et al., 2017; Rouse, Carvajal & Pleijel, 2018). However, only *Parahesione luteola* (Webster, 1879) and *Parahesione* sp. are known from mud shrimp burrows (Pettibone, 1956; Britayev & Antokhina, 2012). *Parahesione* was proposed by Pettibone (1956) for *Podarke luteola* Webster, 1879 (type species), whose type material was lost, and *Hesione agilis* Webster & Benedict, 1884. The former was found on an oyster bank in Great Egg Harbor, New Jersey, whereas the latter was found living commensally with *Upogebia affinis* (Say, 1818). These two species were regarded by Pettibone (1963) as synonyms and have been considered as facultative symbionts (Martin & Britayev, 1998).

The phylogenetic relationships among hesionids are well known, providing an excellent base to assess the evolution of morphological characters (Ruta et al., 2007; Martin et al., 2015; Bonifácio, Lelièvre & Omnes, 2018; Rouse, Carvajal & Pleijel, 2018). However, additional studies are required to understand (1) the nature of their adaptations to a symbiotic mode of life and (2) the evolutionary consequences of their symbiotic relationships with burrowing decapods to try to elucidate their adaptability to differential environmental conditions.

In this article, we describe two new species of Hesionidae living inside burrows of callianassid ghost shrimps and analyze the phylogenetic relationships within the family, based on four concatenated genes, to assess the evolution of both symbiotic species and their adaptations to living inside host burrows.

## Materials and Methods

**Figure 1:**
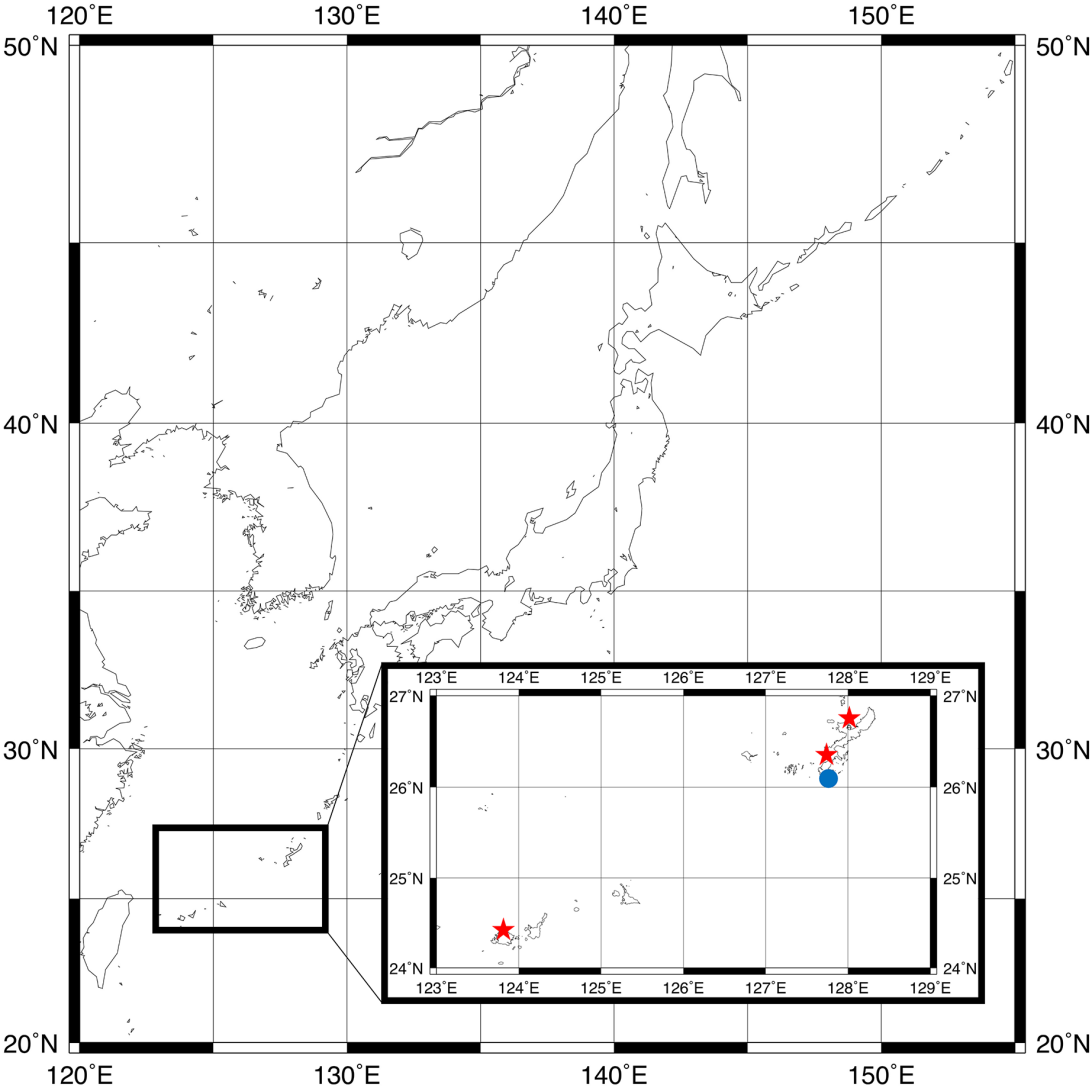
Sampling locations for type specimens. *Parahesione pulvinata* sp. nov. (Red star) and *Parahesione apiculata* sp. nov. (Blue dot). The map was generated using GMT 6 (Wessel et al., 2019; https://www.generic-mapping-tools.org/).

The specimens were collected with a yabby pump from inside of the burrows of *Neocallichirus jousseaumei* (Nobili, 1904) (Axiidea: Callichiridae) and *Glypturus armatus* (Milne-Edwards, 1870) (Axiidea: Callichiridae), living in tidal flats throughout the Ryukyu Islands, Japan (Fig. 1). All specimens were fixed and preserved in 70% ethanol. Additional studied specimens were reported as: (1) *Parahesione* sp. (Britayev & Antokhina, 2012), (2) *Parahesione* sp. (Ruta et al., 2007, first paragraph, page 101), reported as *P*. *luteola* in Genbank, (3) *Parahesione* from Papua New Guinea, collected by GR likely from burrow of *Calliaxina bulimba* (Poore & Griffin, 1979) (Axiidea: Eucalliacidae), and (4) *P*. *luteola* (Pettibone, 1956) (No. USNM 430 and 28175).

The Japanese specimens were observed using a Nikon SMZ1500 dissecting microscope and a Nikon ECLIPSE Ni-U compound light microscope. Photographs were taken with an Olympus OM-D5 digital camera. The Papua New Guinea specimen was observed with a Leica MZ9.5 stereomicroscope and photographed with a Canon Rebel T3i camera.

Type specimens are deposited in the National Museum of Nature and Science, Tsukuba, Japan (NSMT) and the Scripps Institution of Oceanography Benthic Invertebrate Collection, La Jolla, California, USA (SIO-BIC). The following abbreviations are used in the ‘Material examined’ section: CS (complete specimens), L (length, measured from the anterior margin of the prostomium to the posterior border of the last segment), W (width the widest segment, including parapodia but excluding chaetae).

The total DNA of the Japanese holotype was extracted from a dissected parapodium using a DNeasy Tissue Kit (Qiagen, Hilden, Germany). DNA extraction, sequencing, alignment, and removing ambiguous positions were carried out for the method of Jimi et al. (2021). The *Parahesione* from Papua New Guinea were extracted following from Rouse, Carvajal & Pleijel (2018) and *COI* was generated for the specimen. All newly obtained sequences *COI* (625 bp), *16S* (552 bp), *18S* (1,677 bp), *28S* (987 bp) were deposited in GenBank. 198 sequences (59 species) were used for molecular analyses, including 191 downloaded from GenBank (https://www.ncbi.nlm.nih.gov/genbank) (Table 1). Phylogenetic analyses were also carried out for the method of Jimi et al. (2021). *Dysponetus caecus* (Langerhans, 1880) was used as the outgroup following Rouse, Carvajal & Pleijel (2018) and Tilic et al. (2022). Additionally, four species were used for calculating K2P genetic distances using MEGAX (Stecher, Tamura & Kumar, 2020). To understand the evolution of symbiosis in the family Hesionidae, we divided them into the following three groups: obligate and facultative symbionts, according to Martin et al. (2017), and free-living based on previously published data (Table 2). In the molecular phylogenetic tree, we mapped species with symbiotic ecology based on this table.

**Table 1:** List of hesionids included in the phylogenetic analysis and the Genbank accession numbers.

Species	18S	16S	28S	COI	References
*Dysponetus caecus* (Langerhans, 1880)	AY839568	EU555047	EU555028	AF221568	Wiklund et al. (2009), Dahlgren et al. (2000)
*Amphiduros fuscescens* (Marenzeller, 1875)	DQ442584	DQ442569	DQ442598	DQ442561	Ruta et al. (2007)
*Amphiduropsis* cf. *axialensis sensu* Rouse, Carvajal & Pleijel (2018)	MG649239	MG523356	MG649243	MG517505	Rouse, Carvajal & Pleijel (2018)
*Amphiduros pacificus* Hartman (1961)	JN631334	JN631324	JN631345	JN631312	Pleijel et al. (2012)
*Elisesione imajimai* Jimi, Eibye-Jacobsen & Salazar-Vallejo, 2018	LC361352	–	LC361353	LC361354	Jimi, Eibye-Jacobsen & Salazar-Vallejo, 2018
*Gyptis brunnea* (Hartman, 1961)	JN631335	JN631323	JN631346	JN631313	Pleijel et al. (2012)
*Gyptis golikovi* (Averincev, 1990)	JN631336	JN631321	JN631347	–	Pleijel et al. (2012)
*Gyptis hians* Fauchald & Hancock, 1981	JN571891	JN571880	JN571900	JX503009	Summers, Pleijel & Rouse (2015)
*Gyptis pacificus* (Hessle, 1925)	JN631337	JN631322	JN631348	JN631314	Pleijel et al. (2012)
*Gyptis paucilineata* Pleijel, Rouse & Nygren (2009)	–	–	–	EU498243	Pleijel, Rouse & Nygren (2009)
*Gyptis polymorpha* Pleijel, Rouse & Nygren, 2009	–	–	–	EU498247	Pleijel et al. (2012)
*Gyptis propinqua* Marion & Bobretzky, 1875	–	DQ442573	DQ442602	EU498229	Pleijel, Rouse & Nygren (2009)
*Gyptis robertscrippsi* Rouse, Carvajal & Pleijel, 2018	MG649238	MG523360	MG649247	MG517513	Rouse, Carvajal & Pleijel (2018)
*Gyptis simpsonorum* Pleijel, Rouse & Nygren, 2009	–	–	–	KU738203	Pleijel, Rouse & Nygren (2009)
*Gyptis shannonae* Summers, Pleijel & Rouse, 2015	KP745537	KP745534	KP745540	–	Summers, Pleijel & Rouse (2015)
*Hesiolyra bergi* Blake, 1985	AM159577	MG523359	–	MG517521	Rouse, Carvajal & Pleijel (2018)
*Hesione* sp. *sensu* Ruta et al. (2007)	DQ442617	DQ442615	DQ442619	–	Ruta et al. (2007)
*Hesiospina aurantiaca* (Sars, 1862)	JN852829	JN631319	JN852897	–	Pleijel et al. (2012)
*Hesiospina vestimentifera* Blake, 1985	JN631330	JN852860	JN631343	JN631310	Pleijel et al. (2012)
*Heteropodarke formalis* Perkins, 1984	–	KJ855065	KJ855076	–	Martin et al. (2015)
*Heteropodarke pleijeli* Muona, 2006	–	KY823464	KY823481	–	Martin et al. (2015)
*Leocrates chinensis* Kinberg, 1866	DQ442589	DQ442575	DQ442605	DQ442565	Ruta et al. (2007)
*Leocratides kimuraorum* Jimi, Tanaka & Kajihara, 2017	LC480516	LC480518	LC480517	LC258082	Jimi, Tanaka & Kajihara (2017), Goto, Hirabayashi & Palmer (2019)
*Micropodarke dubia* (Hessle, 1925)	JN571888	DQ442576	JN571899	JN571825	Summers, Pleijel & Rouse (2015)
*Neogyptis carriebowcayi* Pleijel et al., 2012	JN631338	JN631325	JN631349	JN631315	Pleijel et al. (2012)
*Neogyptis fauchaldi* Pleijel et al., 2012	JN631339	JN631326	–	JN631316	Pleijel et al. (2012)
*Neogyptis hinehina* Pleijel, et al., 2012	JN631340	JN631328	JN631350	JN631317	Pleijel et al. (2012)
*Neogyptis jeffruoccoi* Rouse, Carvajal & Pleijel, 2018	JN852831	–	MG649244	MG517514	Rouse, Carvajal & Pleijel (2018)
*Neogyptis mediterranea* (Pleijel, 1993)	–	DQ442572	DQ442601	DQ442563	Ruta et al. (2007)
*Neogyptis rosea* (Malm, 1874)	JN571890	DQ442574	DQ442603	JN571826	Ruta et al. (2007), Summers, Pleijel & Rouse (2015)
*Neogyptis julii* Summers, Pleijel & Rouse, 2015	KP745538	KP745535	KP745541	KP745532	Summers, Pleijel & Rouse (2015)
*Neogyptis* sp. A *sensu* Pleijel et al. (2012)	JN631341	JN631327	JN631351	JN631318	Pleijel et al. (2012)
*Nereimyra aphroditoides* (Fabricius, 1780)	–	JF317211	JF317204	JF317198	Pleijel et al. (2012)
*Nereimyra punctata* (Müller, 1788)	DQ442591	DQ442577	DQ442606	DQ442566	Ruta et al. (2007)
*Nereimyra woodsholea* (Hartman, 1965)	–	–	JF317207	AY644802	Nygren, Pleijel & Sundberg (2005)
*Oxydromus fauveli* (Uchida, 2004)	–	–	KJ855078	KJ855071	Martin et al. (2015)
*Oxydromus flexuosus* (Delle Chiaje, 1825)	DQ442592	DQ442578	DQ442607	DQ442567	Ruta et al. (2007)
*Oxydromus okupa* (Martin et al., 2017)	KJ855075	KJ855070	KJ855082	–	Martin et al. (2015, 2017), Meca, Drake & Martin (2019)
*Oxydromus microantennatus* (Hutchings & Murray, 1984)	–	KJ855067	KJ855079	KJ855072	Martin et al. (2015)
*Oxydromus obscurus* (Verrill, 1873)	–	KJ855068	KJ855080	KJ855073	Martin et al. (2015)
*Oxydromus pallidus* Claparède, 1864	DQ442593	DQ442579	DQ442608	–	Ruta et al. (2007)
*Oxydromus pugettensis* (Johnson, 1901)	DQ790086	KJ855069	KJ855081	KJ855074	Martin et al. (2015)
*Parahesione apiculata* sp. nov.	–	OP407586	OP407537	OP404167	This study
*Parahesione pulvinata* sp. nov.	OP407566	OP407585	OP407536	OP404166	This study
*Parahesione* sp.	–	–	DQ442613	–	Ruta et al. (2007)
*Podarkeopsis arenicolus* (La Greca, 1946)	JN571889	JN571879	DQ442609	JN571827	Summers, Pleijel & Rouse (2015)
*Podarkeopsis helgolandicus* (Hilbig & Dittmer, 1979)	JN631331	–	JN631344	JN631311	Pleijel et al. (2012)
*Psamathe fusca* Johnston, 1836	DQ442595	DQ442581	DQ442610	DQ513294	Ruta et al. (2007)
*Sirsoe dalailamai* Rouse, Carvajal & Pleijel, 2018	MG649240	MG523357	MG649245	MG517498	Rouse, Carvajal & Pleijel (2018)
*Sirsoe methanicola* (Desbruyères & Toulmond, 1998)	JN631332	DQ442582	DQ442611	DQ513295	Ruta et al. (2007)
*Sirsoe munki* Rouse, Carvajal & Pleijel, 2018	MG649241	MG523358	MG649246	MG517510	Rouse, Carvajal & Pleijel (2018)
*Sirsoe sirikos* Summers, Pleijel & Rouse, 2015	JN571893	JN571882	JN571902	JN571829	Summers, Pleijel & Rouse (2015)
*Syllidia armata* Quatrefages, 1866	DQ442596	DQ442583	DQ442612	DQ442568	Ruta et al. (2007)
*Vrijenhoekia balaenophila* Pleijel et al., 2008	JN631333	DQ513301	DQ513306	DQ513296	Pleijel et al. (2008)
*Vrijenhoekia ahabi* Summers, Pleijel & Rouse, 2015	JN571898	JN571887	JN571907	JN571876	Summers, Pleijel & Rouse (2015)
*Vrijenhoekia falenothiras* Summers, Pleijel & Rouse, 2015	JN571897	JN571886	JN571906	JN571875	Summers, Pleijel & Rouse (2015)
*Vrijenhoekia ketea* Summers, Pleijel & Rouse, 2015	JN571896	JN571885	JN571905	JN571838	Summers, Pleijel & Rouse (2015)
*Vrijenhoekia* sp. A *sensu* Summers, Pleijel & Rouse, 2015	KP745539	KP745536	KP745542	KP745533	Summers, Pleijel & Rouse (2015)

**Table 2:** Life style of hesionids included in the phylogenetic analysis, indicating the mode of life and the host taxa in case of symbionts.

Species	Mode of life	Host	References
*Dysponetus caecus* (Langerhans, 1880)	Free-living	–	Watson et al. (2014)
*Amphiduros fuscescens* (Marenzeller, 1875)	Free-living	–	Pleijel (2001)
*Amphiduropsis* cf. *axialensis sensu* Rouse, Carvajal & Pleijel (2018)	Free-living	–	Rouse, Carvajal & Pleijel (2018)
*Amphiduros pacificus* Hartman, 1961	Free-living	–	Pleijel (2001)
*Elisesione imajimai* Jimi, Eibye-Jacobsen & Salazar-Vallejo, 2018	Free-living	–	Jimi, Eibye-Jacobsen & Salazar-Vallejo (2018)
*Gyptis brunnea* (Hartman, 1961)	Free-living	–	Hartman (1961)
*Gyptis golikovi* (Averincev, 1990)	Free-living	–	Averincev (1990)
*Gyptis hians* Fauchald & Hancock, 1981	Free-living	–	Banse & Hobson (1968)
*Gyptis pacificus* (Hessle, 1925)	Free-living	–	Hessle (1925)
*Gyptis paucilineata* Pleijel, Rouse & Nygren, 2009	Free-living	–	Pleijel, Rouse & Nygren (2009)
*Gyptis polymorpha* Pleijel, Rouse & Nygren, 2009	Free-living	–	Pleijel, Rouse & Nygren (2009)
*Gyptis propinqua* Marion & Bobretzky, 1875	Free-living	–	Parapar, Besteiro & Moreira (2005)
*Gyptis robertscrippsi* Rouse, Carvajal & Pleijel, 2018	Free-living	–	Rouse, Carvajal & Pleijel (2018)
*Gyptis simpsonorum* Pleijel, Rouse & Nygren, 2009	Free-living	–	Pleijel, Rouse & Nygren (2009)
*Gyptis shannonae* Summers, Pleijel & Rouse, 2015	Free-living	–	Summers, Pleijel & Rouse (2015)
*Hesiolyra bergi* Blake, 1985	Free-living	–	Blake (1985)
*Hesione* sp. *sensu* Ruta et al. (2007)	Free-living	–	Ruta et al. (2007)
*Hesiospina aurantiaca* (Sars, 1862)	Free-living	–	Pleijel (2004)
*Hesiospina vestimentifera* Blake, 1985	Facultative symbiont	Annelids	Pleijel (2004)
*Heteropodarke formalis* Perkins, 1984	Free-living	–	Perkins (1984)
*Heteropodarke pleijeli* Muona, 2006	Free-living	–	Pleijel (1999), Muona (2006)
*Leocrates chinensis* Kinberg, 1866	Facultative symbiont	Corals	Martin et al. (2017), Wang, Qiu & Salazar-Vallejo (2018)
*Leocratides kimuraorum* Jimi, Tanaka & Kajihara, 2017	Obligate-symbiont	Sponges	Jimi, Tanaka & Kajihara (2017)
*Micropodarke dubia* Hessle (1925)	Free-living	–	Hessle (1925)
*Neogyptis carriebowcayi* Pleijel et al., 2012	Free-living	–	Pleijel et al. (2012)
*Neogyptis fauchaldi* Pleijel et al., 2012	Free-living	–	Pleijel et al. (2012)
*Neogyptis hinehina* Pleijel et al. (2012)	Free-living	–	Pleijel et al. (2012)
*Neogyptis jeffruoccoi* Rouse, Carvajal & Pleijel, 2018	Facultative symbiont	Bivalves	Rouse, Carvajal & Pleijel (2018)
*Neogyptis mediterranea* (Pleijel, 1993)	Free-living	–	Pleijel et al. (2012)
*Neogyptis rosea* (Malm, 1874)	Free-living	–	Pleijel et al. (2012)
*Neogyptis julii* Summers, Pleijel & Rouse, 2015	Free-living	–	Summers, Pleijel & Rouse, 2015
*Neogyptis* sp. A *sensu* Pleijel et al. (2012)	Free-living	–	Pleijel et al. (2012)
*Nereimyra aphroditoides* (Fabricius, 1780)	Free-living	–	Pleijel, Rouse & Nygren (2011)
*Nereimyra punctata* (Müller, 1788)	Free-living	–	Pleijel, Rouse & Nygren (2011)
*Nereimyra woodsholea* (Hartman, 1965)	Free-living	–	Pleijel, Rouse & Nygren (2011)
*Oxydromus fauveli* Uchida, Lopez & Sato, 2019	Free-living	–	Uchida, Lopez & Sato (2019)
*Oxydromus flexuosus* (Delle Chiaje, 1825)	Facultative symbiont	Starfish, holothuroids, annelids	Martin et al. (2017)
*Oxydromus humesi* (Pettibone, 1961)	Obligate symbiont	Bivalves	Pettibone (1961), Martin et al. (2012, 2017)
*Oxydromus okupa* (Martin et al., 2017)	Obligate symbiont	Bivalves	Martin et al. (2012, 2017), Meca, Drake & Martin (2019)
*Oxydromus microantennatus* (Hutchings & Murray, 1984)	Free-living	–	Hutchings & Murray (1984)
*Oxydromus obscurus* (Verrill, 1873)	Facultative symbiont	Annelids, holothuroids	Martin & Britayev (1998)
*Oxydromus pallidus* Claparède, 1864	Facultative symbiont	Annelids	Martin et al. (2017)
*Oxydromus pugettensis* (Johnson, 1901)	Facultative symbiont	Echinoderms, decapods, gastropods, bivalves	Martin & Britayev (1998)
***Parahesione apiculata* sp. nov.**	Obligate symbiont	Decapods	This study
***Parahesione pulvinata* sp. nov.**	Obligate symbiont	Decapods	This study
*Parahesione* sp.	Facultative symbiont?	Decapods	Ruta et al. (2007)
*Podarkeopsis arenicolus* (La Greca, 1946)	Free-living	–	La Greca (1946)
*Podarkeopsis helgolandicus* (Hilbig & Dittmer, 1979)	Free-living	–	Hilbig & Dittmer (1979)
*Psamathe fusca* Johnston, 1836	Free-living	–	Parapar, Besteiro & Moreira (2005)
*Sirsoe dalailamai* Rouse, Carvajal & Pleijel, 2018	Free-living	–	Rouse, Carvajal & Pleijel (2018)
*Sirsoe methanicola* (Desbruyères & Toulmond, 1998)	Free-living	–	Desbruyères & Toulmond (1998)
*Sirsoe munki* Rouse, Carvajal & Pleijel, 2018	Free-living	–	Rouse, Carvajal & Pleijel (2018)
*Sirsoe sirikos* Summers, Pleijel & Rouse, 2015	Free-living	–	Summers, Pleijel & Rouse (2015)
*Syllidia armata* Quatrefages, 1866	Free-living	–	Ruta & Pleijel (2006)
*Vrijenhoekia balaenophila* Pleijel et al., 2008	Free-living	–	Pleijel et al. (2008)
*Vrijenhoekia ahabi* Summers, Pleijel & Rouse, 2015	Free-living	–	Summers, Pleijel & Rouse (2015)
*Vrijenhoekia falenothiras* Summers, Pleijel & Rouse, 2015	Free-living	–	Summers, Pleijel & Rouse (2015)
*Vrijenhoekia ketea* Summers, Pleijel & Rouse, 2015	Free-living	–	Summers, Pleijel & Rouse (2015)
*Vrijenhoekia* sp. A *sensu* Summers, Pleijel & Rouse (2015)	Free-living	–	Summers, Pleijel & Rouse (2015)

The map of Fig. 1 was generated by using GMT 6 (Wessel et al., 2019).

The electronic version of this article in Portable Document Format (PDF) will represent a published work according to the International Commission on Zoological Nomenclature (ICZN), and hence the new names contained in the electronic version are effectively published under that Code from the electronic edition alone. This published work and the nomenclatural acts it contains have been registered in ZooBank, the online registration system for the ICZN. The ZooBank LSIDs (Life Science Identifiers) can be resolved and the associated information viewed through any standard web browser by appending the LSID to the prefix http://zoobank.org/. The LSID for this publication is: urn:lsid:zoobank.org:pub:6D64D9F4-0E29-4F67-B941-300E1888108C. The online version of this work is archived and available from the following digital repositories: PeerJ, PubMed Central SCIE and CLOCKSS.

## Results

### Systematics

**Ophiodrominae Pleijel, 1998**

**Amphidurini Pleijel et al., 2012**

***Parahesione* Pettibone, 1956**

**Diagnosis (emended).** Body depressed, reddish when alive. Prostomium with two lateral antennae, without median antenna, two pairs of eyes. Palps simple or biarticulate. Six or eight pairs of tentacular cirri. Dorsal cirrophores fused with or without dorsal foliose lobe extending to base of parapodia. Parapodia biramous. Notopodia with numerous capillary chaetae. Neuropodia with numerous compound chaetae: homogomph and/or heterogomph falcigers, and heterogomph spinigers (after Pettibone, 1956).

**Remarks.** *Parahesione* resembles *Amphiduros* and *Amphiduropsis* in having enlarged dorsal cirri on segments 1–5, but differs in lacking median antenna (having a short one in *Amphiduros* and *Amphiduropsis*). Two species of the genus, type species *P. luteola* and *Parahesione* sp. from New Caledonia (Ruta et al., 2007) have six enlarged tentacular cirri and cilindrical dorsal cirrophores, while two new species, *P. pulvinata* and *P. apiculata* have eight pairs of tentacular cirri and dorsal cirrophores fused with dorsal foliose lobe extending to base of parapodia. We assign these species to the same genus *Parahesione* and modify the diagnosis of the genus. However, since the DNA repository data for *Parahesione* sp. used in Ruta et al. (2007) is very limited, and for the type species *P. luteola* is unavailable (formaldehyde fixation), it is possible that the two morpho-types *Parahesione* (eight enlarged cirri & dorsal cirrophores with dorsal foliose lobe *vs*. six enlarged cirri & dorsal cirrophores without dorsal foliose lobe) would be assigned to different genera if molecular sequences for additional specimens of first morpho-type would be obtained.

***Parahesione pulvinata*** Jimi, Gonzalez, Rouse and Britayev sp. nov.

**Figure 2:**
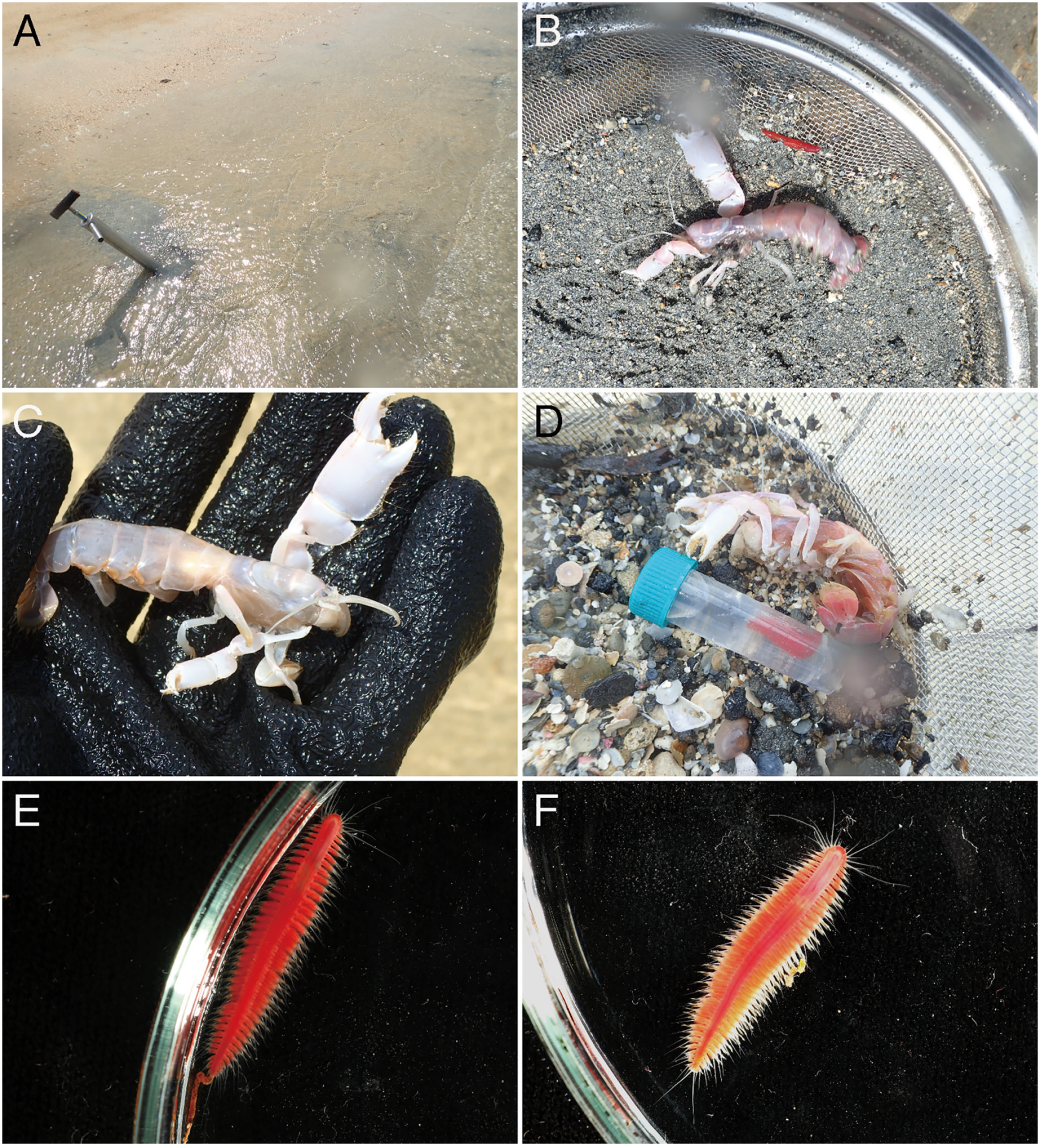
Observations of *Parahesione pulvinata* sp. nov. and its hosts *in situ*. (A) Sampling at the sandy tidal flat of Uehara; (B) host and the new species; (C) detail of the host *Neocallichirus jousseaumei*; (D) another host with its symbiont (tube); (E) dorsal view of the new species of a living specimen (NSMT-Pol H-893); (F) dorsal view of a preserved specimen (NSMT-Pol H-893).

**Figure 3:**
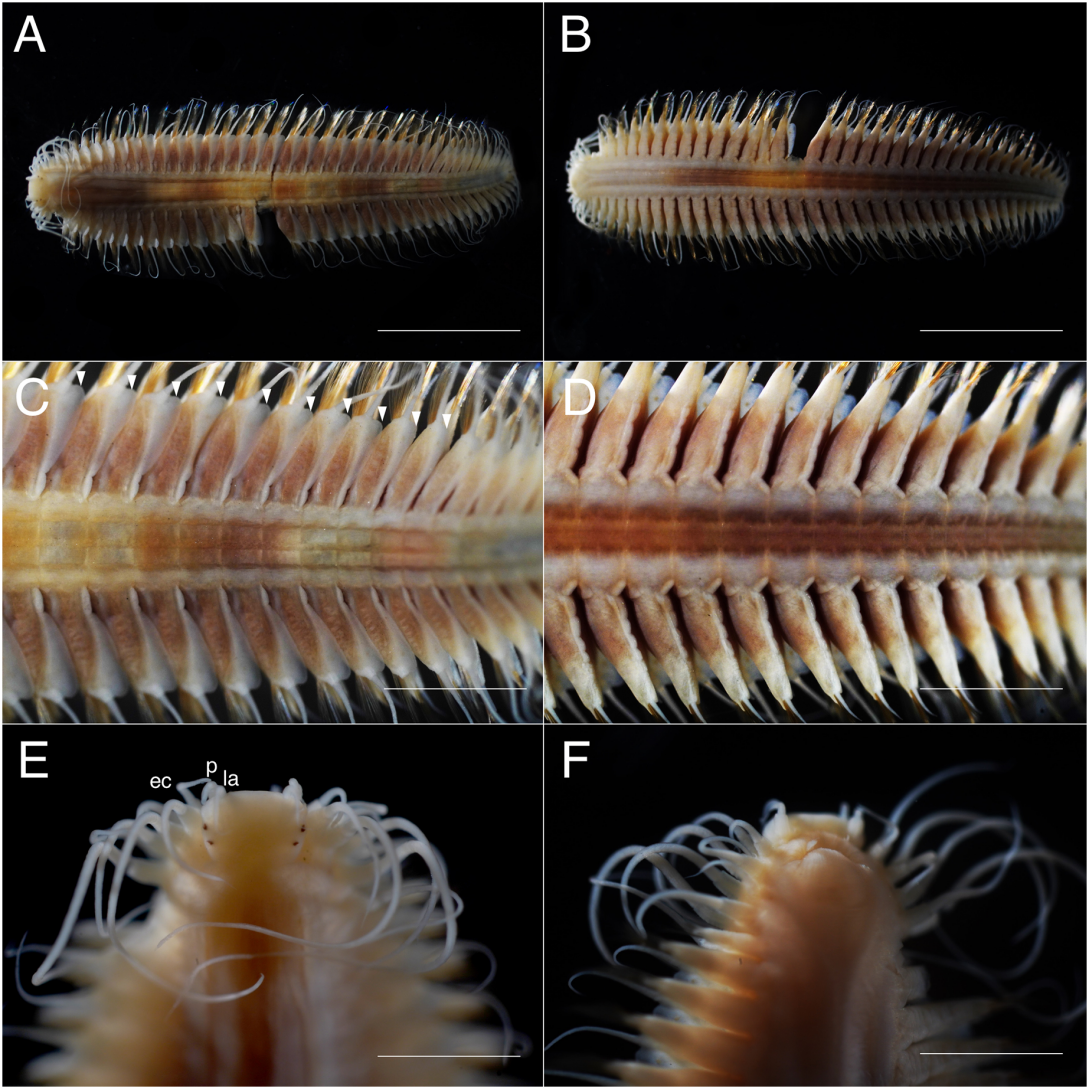
*Parahesione pulvinata* sp. nov. (NSMT-Pol H-893). (A) Whole specimen, dorsal view; (B) whole specimen, ventral view; (C) middle segments, dorsal view; (D) middle segments, ventral view; (E) anterior end, dorsal view; (F) anterior end, ventral view. White arrows indicate pillow-shaped dorsal cirrophore without digitate lobes. Abbreviation: la, lateral antenna; p, palp; ec, enlarged cirrus. Scale bars: A and B, 5 mm; C and D, 2 mm; E and F, 1 mm.

**Figure 4:**
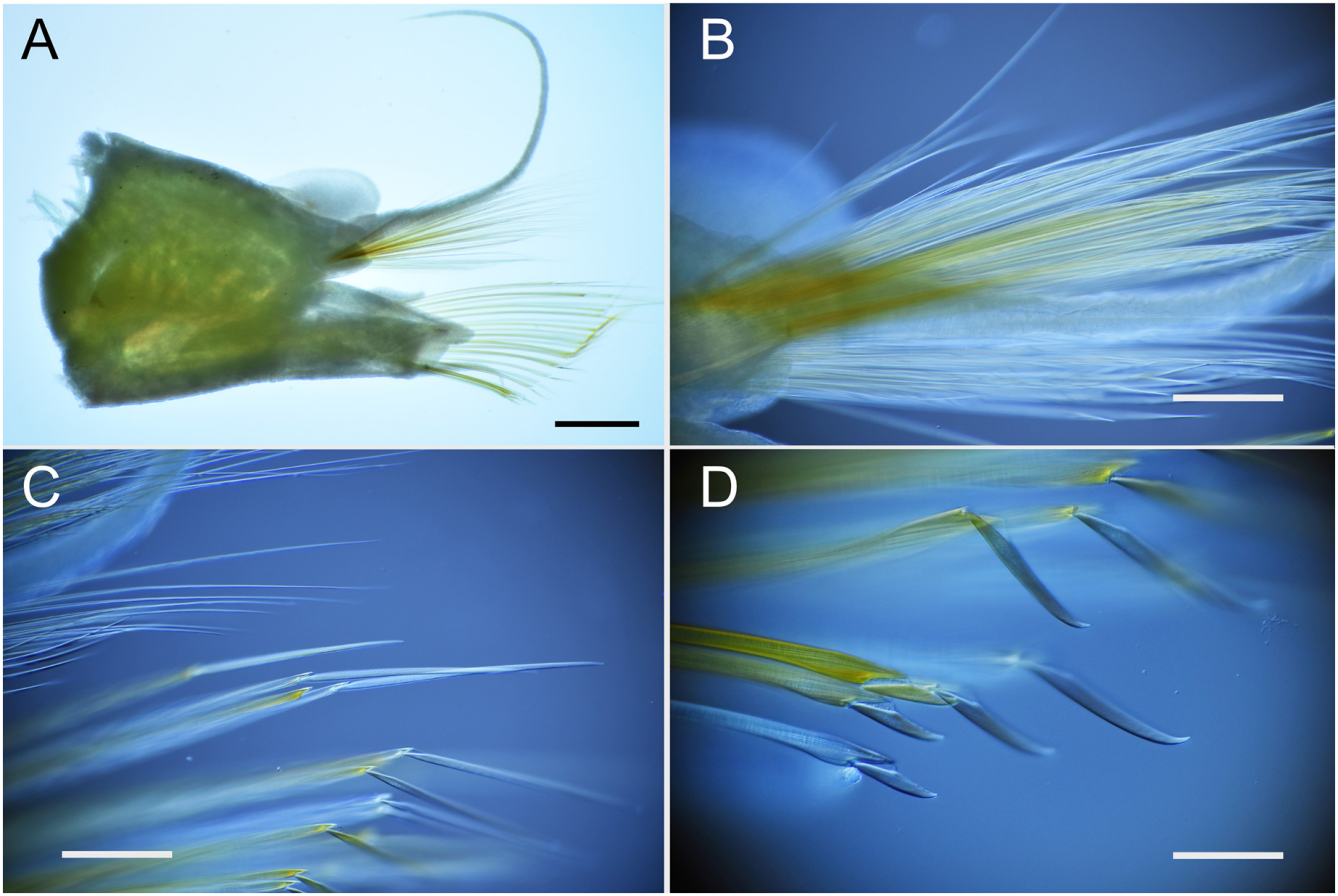
*Parahesione pulvinata* sp. nov. (NSMT-Pol H-893). (A) Parapodium of chaetiger 12, rear view; (B) notochaetae, chaetiger 12; (C) upper side of neurochaetae, chaetiger 12; (D) lower side of neurochaetae. Scale bars: A, 200 μm; B–D, 100 μm.

**Figure 5:**
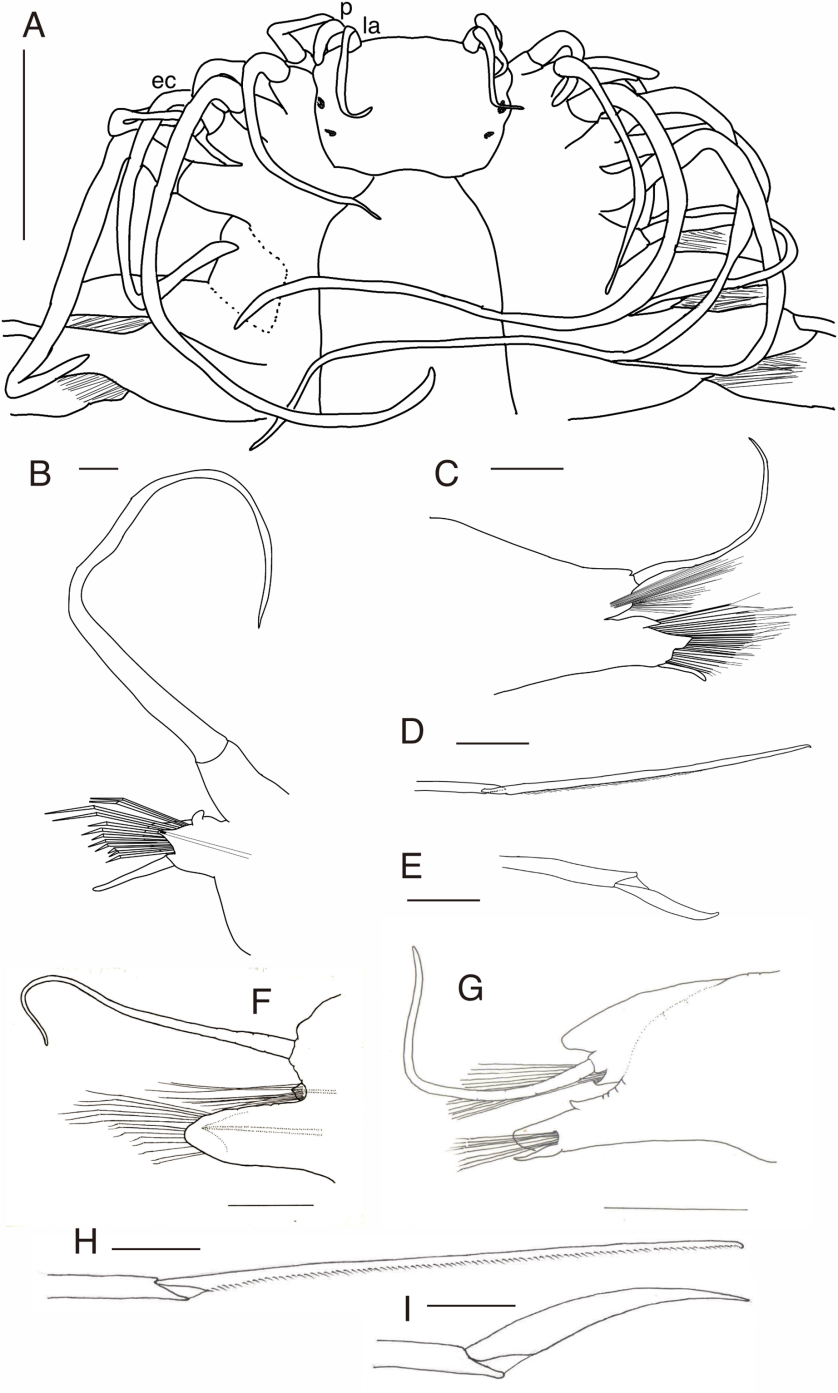
*Parahesione pulvinata* sp. nov. (NSMT-Pol H-893: A–E, IPEE RAS – Pol. 2004/01: E–I). (A) Anterior end, dorsal view; (B) parapodium of chaetiger 1, rear view; (C) parapodium of chaetiger 17, frontal view; (D) neurochaeta, upper side, chaetiger 17; (E) neurochaeta, lower side, chaetiger 17; (F) parapodium of chaetiger 18, frontal view; (G) parapodium of chaetiger 23, rear view; (H) supreacicular spiniger; (I) uppermost falciger. Scale bars: A, 1 mm; B, 100 μm; C, 500 μm; D and E, 100 mm; F and G, 500 μm; H and I, 100 μm. Abbreviation: la, lateral antenna; p, palp; ec, enlarged cirrus.

[New Japanese name: ana-yadori-otohime]

(Figs. 2–5, Fig. S1)

Zoobank LSID: urn:lsid:zoobank.org:act:2E42DB94-DF8C-447A-A7F8-8C2FDA9FF4CA

*Parahesione* sp.: Britayev & Antokhina (2012): 33, Pl. 9 C, D

**Diagnosis.** *Parahesione* with dorsal foliose lobe, without dorso-lateral digitate extension, and eight tentacular anterior cirri.

**Material examined.** Holotype: NMST-Pol H-893, Genbank No.: COI OP404166, 16S OP407585, 18S OP407566, 28S OP407536, CS, L18 mm, W 4 mm for 45 chaetigers, East China Sea, Iriomote Island, Uehara, intertidal area, associated to *N*. *jousseaumei*, 5 September 2020, collected by HN. Paratypes: NSMT-Pol P-894, CS, L 20 mm, W 5 mm for 45 chaetigers, same collection data as holotype, but from another burrow of *N. jousseaumei*. Paratypes: NSMT-Pol P-895, CS, L 17 mm, W 4 mm for 39 chaetigers, East China Sea, Iriomote Island, Uehara, Todomari Beach, 1–2 m in depth, associated to an unknown crustacean, 24 January 2021, collected by TS. Paratypes: NSMT-Pol P-896, CS, L 18 mm, W 4 mm for 36 chaetigers, East China Sea, Okinawa Island, Sunabe, intertidal, associated to an unknown crustacean, 20 September 2021, collected by TS. Paratypes: NSMT-Pol P-897, CS, L 12 mm, W 3 mm for 24 chaetigers, East China Sea, Okinawa Island, Kouri, intertidal, associated to an unknown crustacean, 27 February 2021, collected by HN. SIO-BIC A13742, 1 specimen, Madang Lagoon, Tab Island, Madang Province, Papua New Guinea, 5.17°S; 145.84°E, likely associated to *C. bulimba*, 13 December 2012, collected by Art Anker and GR. Additional material: IPEE RAS—Pol. 2004/01, 1 specimen in four fragments, L 19.5 mm, W 4.4 mm for 48 chaetigers, South China Sea, Nhatrang Bay, River Be estuary, intertidal, sandy silt, associated to *Upogebia* sp., 18 April 2004, collected by Ivan Marin.

**Description of holotype.** Body depressed, tapered in posterior region, reddish when alive (Fig. 2), pinkish after fixation (Fig. 3). Prostomium rectangular, wider than long (Figs. 3E and 5A). Lateral antennae slightly shorter than head length, cylindrical, with distally tapering style and short cylindrical ceratophores. Palps 2/3 antennae length, with cylindrical distally tapering palpostyles and short cylindrical palpophores. Two pairs of eyes, dark reddish in alive, brownish after fixation.

Elongated dorsal cirri on segments 1–5; tentacular cirri eight pairs, on segments 1–4, cirrophores of tentacular cirri cylindrical, basally fused; longest dorsal cirri reaching chaetiger 8, longest ventral cirri reaching chaetiger 5. Chaetae absent from segments 1–4.

Dorsal cirrophores from segment 6 (= chaetiger 2) fused with dorsal foliose lobe extending to base of parapodia, partially covering subsequent segment (Fig. 3C); all dorsal cirrostyles long, twice as long or longer than neuropodial lobe with chaetae, conical, smooth (Fig. 5C). Ventral cirrophores fused to parapodia; ventral cirrostyles short, slightly extending beyond neuropodial lobe, conical, smooth (Figs. 3D and 5C). Noto- and neuro aciculae brownish, tip of aciculae not seen *in vivo*, reddish after fixation.

All chaetigers biramous (Fig. 4A) except chaetiger 1 (uniramous notopodia small, conical, pointed, with about 40 simple capillary very fine notochaetae Fig. 4B); neuropodia large, truncated, longer than wide, with prechaetal lobes and a postero-dorsal digitiform projections (Fig. 5C) and about 30 compound heterogomph chaetae, supraacicular spinigers (Fig. 4C) and subacicular falcigers (Fig. 4D) with unidentate blades faintly serrated in spinigers and superior falcigers (Figs. 5H and 5I); smooth in most inferior falcigers; length of blades in bundle decreases from superior to inferior neurochaetae (Figs. 5D and 5E). Pygidium with two smooth anal cirri twice as long as dorsal cirri.

**Variation.** Body length 12.0–19.5 mm; number of chaetigers 24–48. Morphology of paratypes corresponds to description of holotype; anterior pair of dark red eyes was visible in specimen from Nhatrang when alive.

**Etymology.** The specific name “pulvinata”, derived from the Latin *pulvinus* (meaning cushion, pillow), referring to the shape of dorsal cirrophores. The specific name is an adjective in the nominative case.

**Remarks.** *Parahesione pulvinata* sp. nov. resembles *P*. *luteola*, the type species of the genus and the single previously known species, in lacking the median antenna while having a flattened body and living symbiotically with ghost shrimps. However, it differs in having flattened dorsal parapodial extension and eight tentacular anterior cirri (without extension and and six tentacular cirri in *P*. *luteola*). *Parahesione* sp. from Vietnam (Figs. 5F–5I) and Papua New Guinea (Fig. S1) are morphologically identical to the Japanese materials, therefore confirming that they belong to *P. pulvinata* sp. nov. The COI sequences for the Japanese and Papua New Guinea were only slightly divergent.

**Distribution and habitat.** Ryukyu Islands (Japan, East China Sea), Nhatrang Bay (Vietnam, South China Sea), and Madang Lagoon, Papua New Guinea (Southwestern Pacific Ocean), in intertidal mud flats, living inside burrows of *N. jousseaumei* (Japan) and *Upogebia* sp. (Vietnam), or at 1–5 m inside burrows of *C. bulimba*.

***Parahesione apiculata*** Jimi, Gonzalez, Rouse and Britayev sp. nov.

[New Japanese name: toge-ana-yadori-otohime]

(Figs. 6–9)

**Figure 6:**
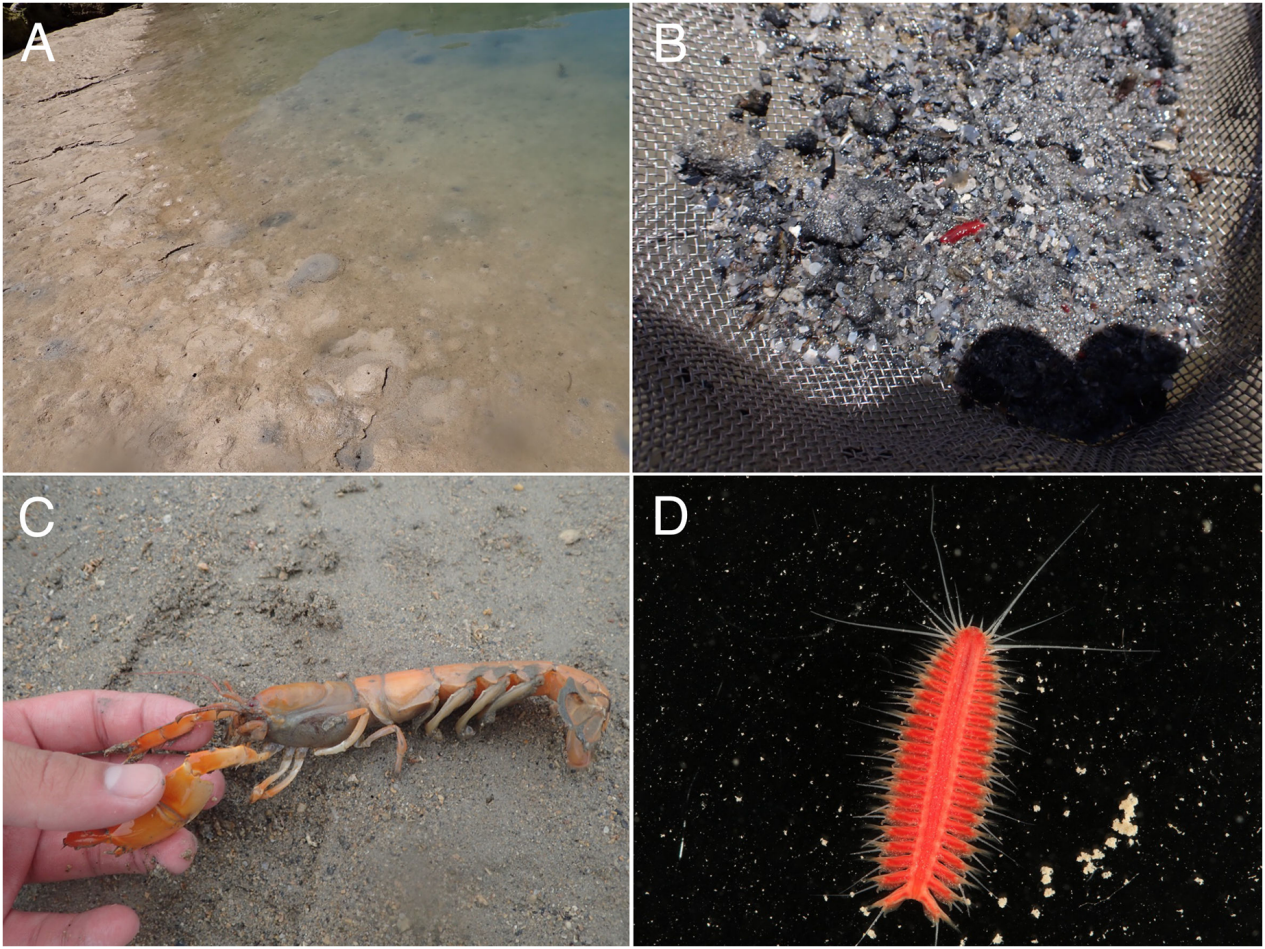
*Parahesione apiculata* sp. nov. and hosts *in situ* (A) Sampling location at the Nanjo sandy tidal flat; (B) living specimen of the symbiont; (C) living specimen of the *Glypturus armatus* (host); (D) dorsal view of a living specimen, lacking posterior most segments (same individual with Fig. 6B, NSMT-Pol P-899).

**Figure 7:**
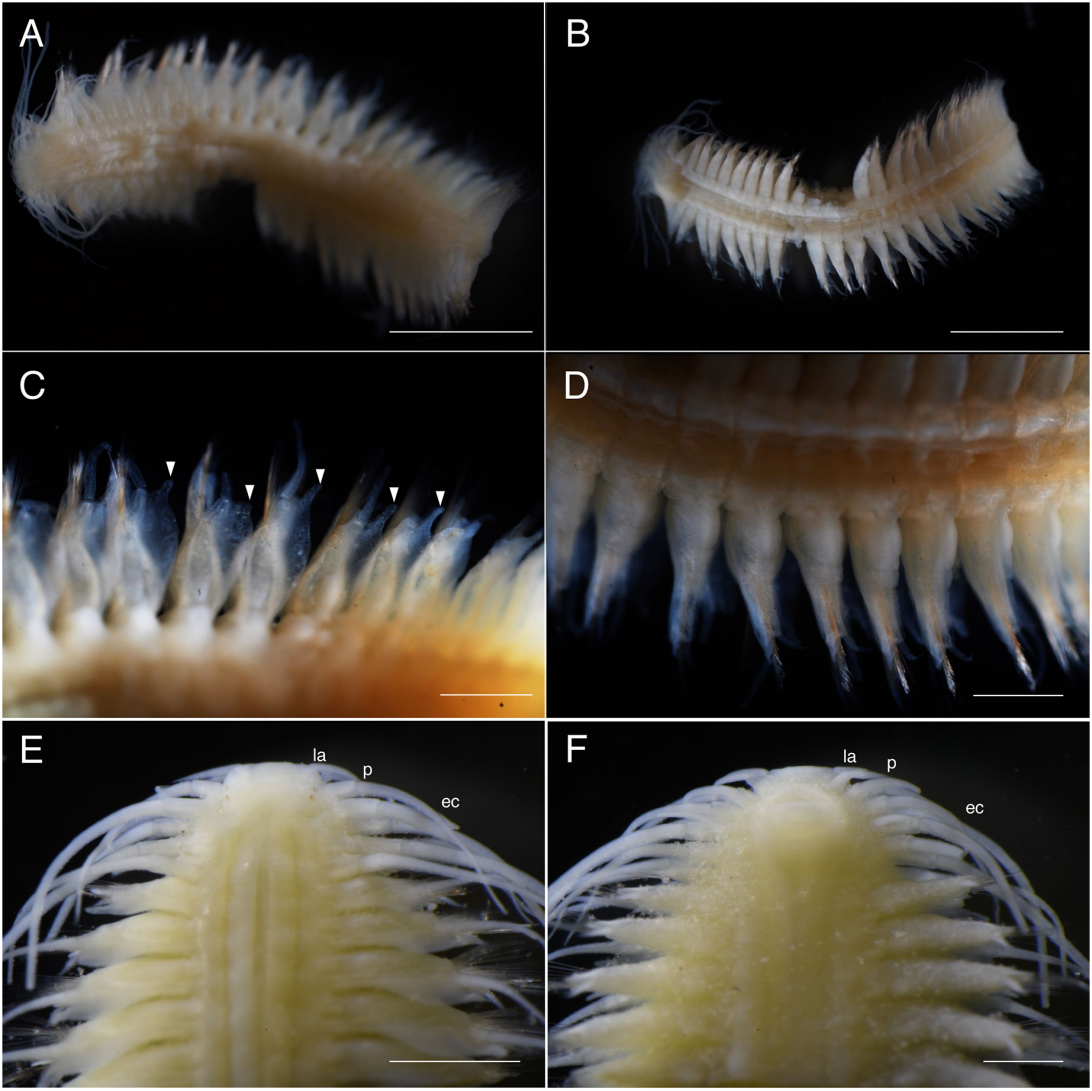
*Parahesione apiculata* sp. nov. (NSMT-Pol H-898). (A) Whole specimen, dorsal view; (B) whole specimen, ventral view; (C) middle segments, dorsal view; (D) middle segments, ventral view; (E) anterior end, dorsal view; (F) anterior end, ventral view. White arrows indicate digitate lobes. Abbreviation: la, lateral antenna; p, palp; ec, enlarged cirrus. Scale bars: A and B, 3 mm; C and D, 1 mm; E and F, 1 mm.

**Figure 8:**
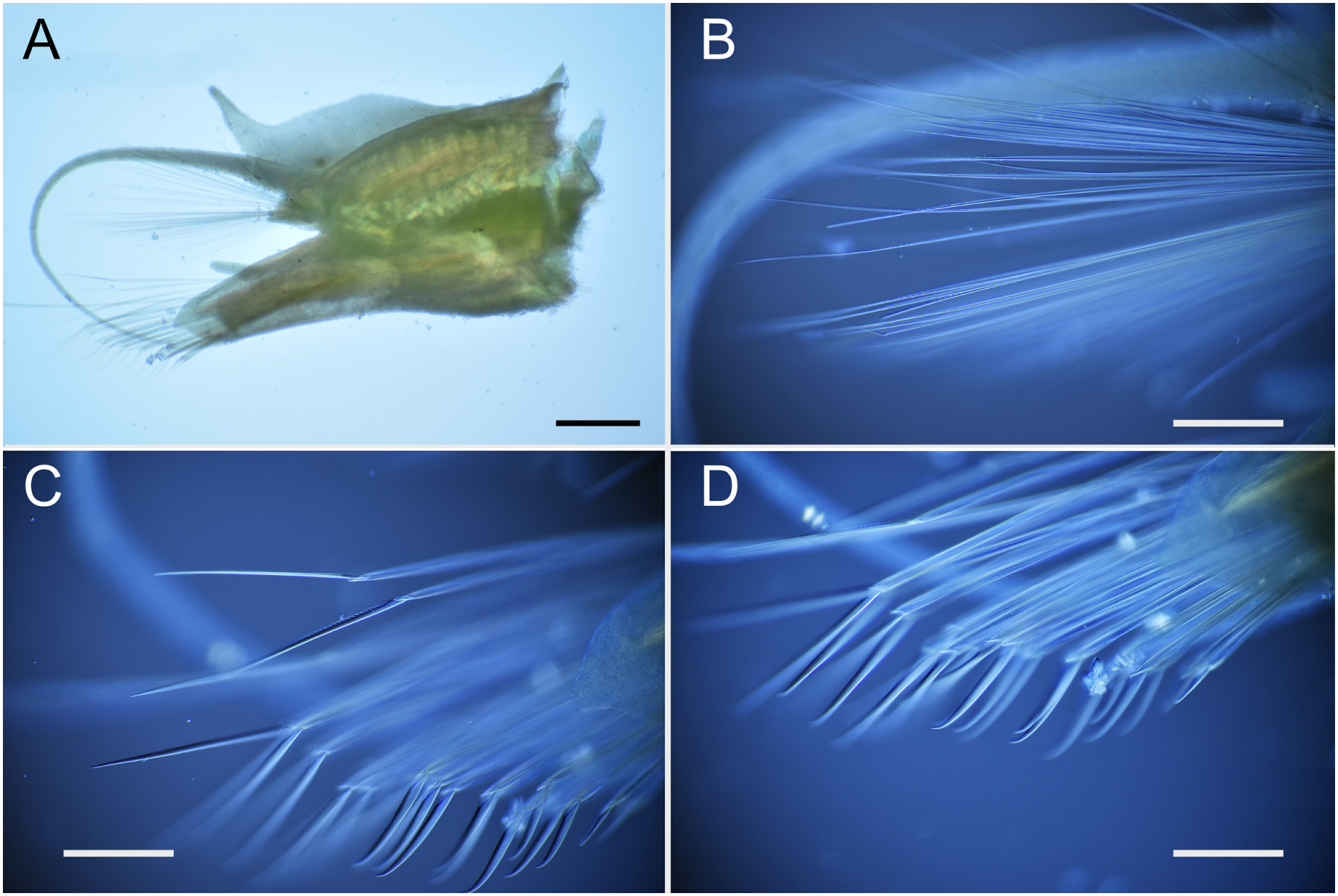
*Parahesione apiculata* sp. nov. (NSMT-Pol H-898). (A) Parapodium of chaetiger 12, frontal view; (B) notochaetae, chaetiger 12; (C) upper side of neurochaetae, chaetiger 12; (D) lower side of neurochaetae. Scale bars: A, 200 μm; B–D, 100 μm.

**Figure 9:**
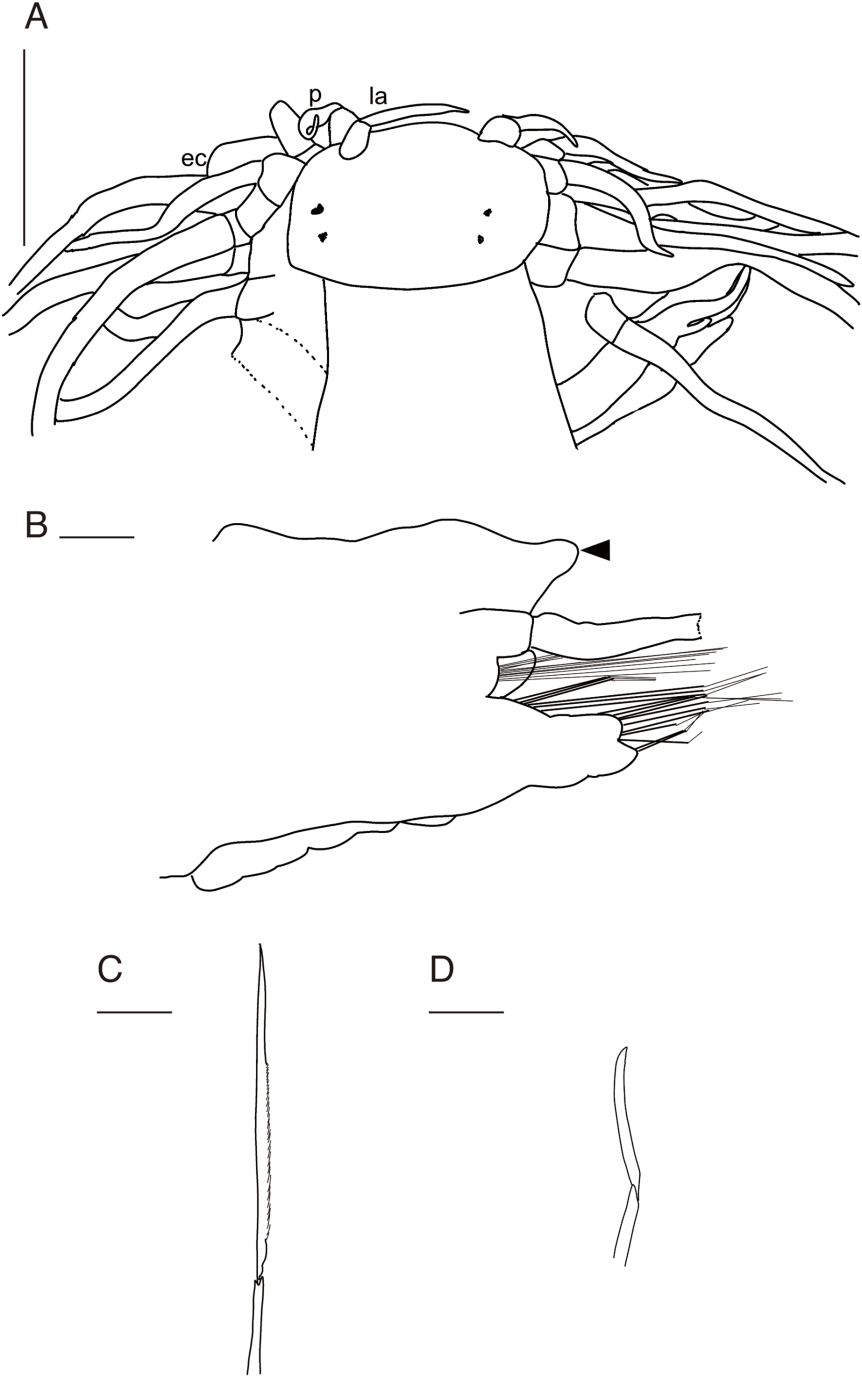
*Parahesione apiculata* sp. nov. (NSMT-Pol H-898). (A) Anterior end, dorsal view; (B) parapodium of chaetiger 17, frontal view; (D) neurochaeta, upper side, chaetiger 17; (D) neurochaeta, lower side, chaetiger 17. Black arrow indicates a digitate lobe. Abbreviation: la, lateral antenna; p, palp; ec, enlarged cirrus. Scale bars: A, 1 mm; B, 500 μm; C and D, 100 mm.

Zoobank LSID: urn:lsid:zoobank.org:act:1AB8DAA4-2268-445D-A3A6-9AE9C085A856

**Diagnosis.** *Parahesione* with dorsal foliose lobe, dorso-lateral digitate extension, and eight tentacular anterior cirri.

**Material examined.** Holotype: NSMT-Pol H-898, Genbank No.: COI OP404167, 16S OP407586, 28S OP407537, specimen with posterior part lost, L 12 mm, W 4 mm for 28 chaetigers, Philippine Sea, Okinawa Island, Shikenbaru beach, 1–2 m in depth, burrow of *G. armatus*, 23 December 2019, collected by TS. Paratype: NSMT-Pol P-899, specimen with posterior part lost, L 8 mm, W 4 mm for 20 chaetigers, Philippine Sea, Okinawa Island, Nanjo, Ou beach, intertidal, burrow of *G. armatus*, 20 August 2021, collected by HN. Paratype: NSMT-Pol P-900, specimen with posterior part lost, L 9 mm, W 3 mm for 24 chaetigers, East China Sea, Okinawa Island, Kujuzaki, intertidal, burrow of *G. armatus*, 22 August 2021, collected by TS.

**Description of holotype.** Body depressed, tapering in posterior region, reddish when alive, pale orange after fixation (Figs. 6 and 7).

Prostomium rectangular, wider than long (Fig. 9A). Lateral antennae as long as head length, cylindrical, with distally tapering style and short cylindrical ceratophores. Palps 5/7 antennae length, with cylindrical distally tapering palpostyles and short cylindrical palpophores. Two pairs of eyes (Figs. 7E and 9A), inconspicuous when alive (Fig. 6D), brownish after fixation (Fig. 7E).

Elongated dorsal cirri on segments 1–5; tentacular cirri eight pairs, on segments 1–4, cirrophores of tentacular cirri cylindrical, basally fused; longest dorsal cirri reaching chaetiger 8, longest ventral cirri reaching chaetiger 7. Chaetae absent from segments 1–4.

All dorsal cirrophores cylindrical; from segment 6 (= chaetiger 2) fused with dorsal foliose lobe extending to base of parapodia, partially covering subsequent segment (Figs. 7C, 8A and 9B); dorsal foliose lobe with dorso-lateral digitate extension; dorsal cirrostyle long, equal, or slightly longer than neuropodia with chaetae, conical, smooth (Fig. 7C). Ventral cirrophore fused with parapodia; ventral cirrostyle short, slightly extending beyond neuropodial lobe, conical, smooth. Noto- and neuroaciculae not seen *in vivo*, brownish with reddish tips when preserved.

All chaetigers biramous except chaetiger 1 (uniramous). Notopodia small, conical, with about 40 simple capillary notochaetae (Fig. 8B), faintly serrated. Neuropodia large, truncated, longer than wide, with pre- and post-chaetal lobes and a digitiform projection present on postero-dorsal part (Figs. 7C and 9B). About 30 heterogomph chaetae, supraacicular spinigers (Fig. 8C) and subacicular falcigers (Fig. 8D) with unidentate blades; faintly serrated in spinigers and superior falcigers; smooth in most inferior falcigers; length of blades in bundle decreases from superior to inferior neurochaetae (Figs. 9C and 9D). Pygidium with two long anal cirri, smooth.

**Etymology.** The specific name “*apiculata”*, derives from the Latin *apiculatus* (meaning short pointed) and referring to the digitate extension on dorso-lateral margin of dorsal foliose lobe, is as an adjective in the nominative case.

**Remarks.** Like *P*. *pulvinata* sp. nov., *P*. *apiculata* sp. nov. resemble *P*. *luteola* in lacking the median antenna, having a flattened body and living symbiotically with ghost shrimps, while differing in having dorsal foliose lobe and eight tentacular anterior cirri (absent and six in *P*. *luteola*). *Parahesione apiculata* sp. nov. differs from *P*. *pulvinata* sp. nov. in having digitate extension on posterior margin of dorsal foliose lobe (digitate extension absent in *P*. *pulvinata* sp. nov.), as well as in living in association with *G. armatus* (*N*. *jousseaumei, C. bulima* and *Upogebia* sp. in *P*. *pulvinata* sp. nov.).

**Distribution and habitat.** Ryukyu Islands (Japan, Philippine Sea and East China Sea), in intertidal mud flats, living inside burrows of *G. armatus*.

### Key to species of *Parahesione*

1. Parapodia with dorsal foliose lobe ……………2 – Parapodia without dorsal foliose lobe*P*. *luteola* (Webster, 1879)
2. Dorsal foliose lobe with digitate extension in opposite side of body ……………………*P*. *apiculata* sp. nov. This study
3. Dorsal foliose lobe without digitate extension in opposite side of body …………………*P*. *pulvinata* sp. nov. This study

### Molecular analyses

**Figure 10:**
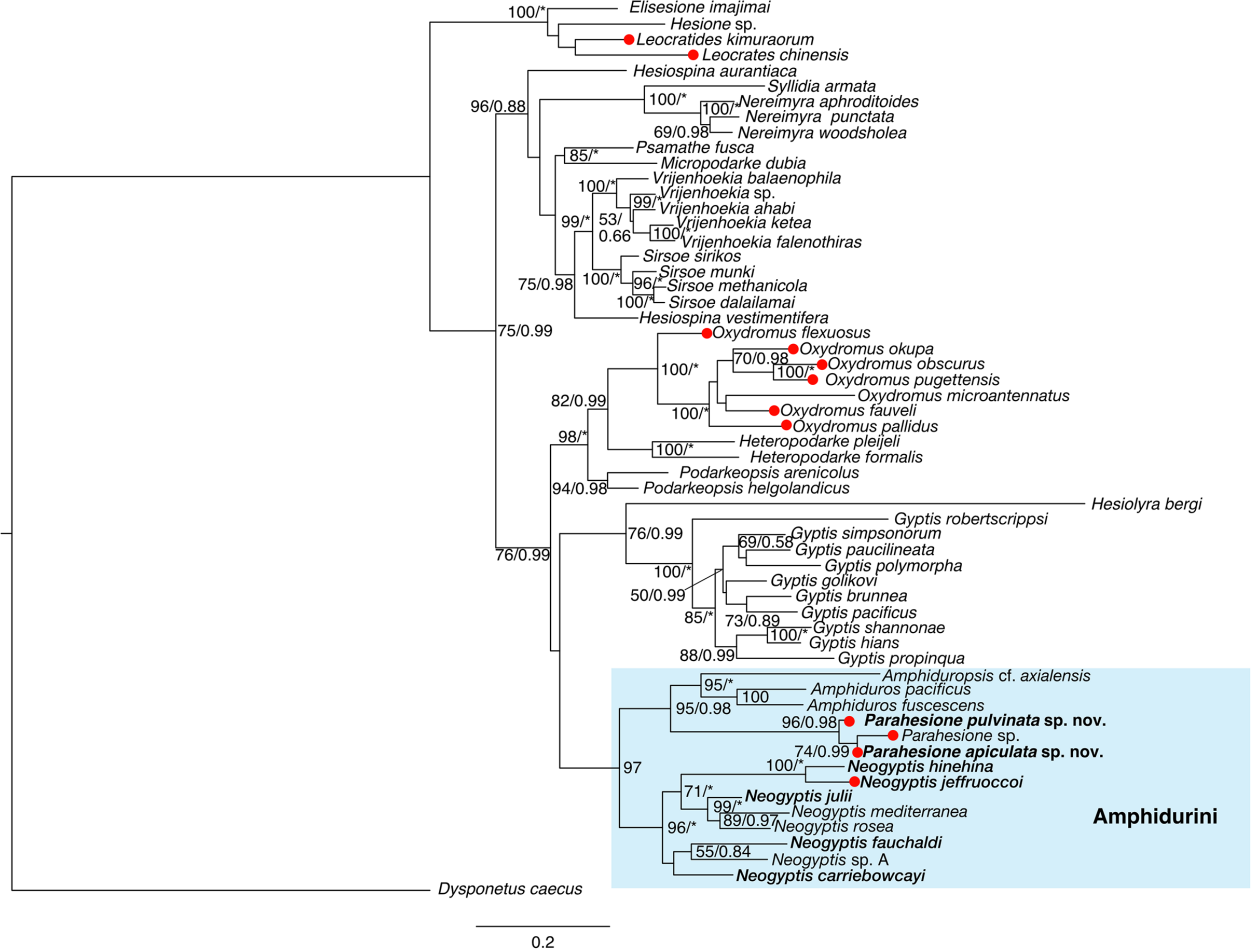
Maximum-likelihood phylogenetic tree of Hesionidae based on *COI*, *16S*, *18S* and *28S* sequences. Nodal bootstrap support (BS) values higher than 50% are indicated for each branch. Posterior probability (PP) of each branch is also shown behind the bootstrap value. * = 100 in BS and 1.00 in PP; - = node absent in the Bayesian tree. Red circles indicate symbiotic species.

*Parahesione apiculata* sp. nov. and *Parahesione* sp. from Papua New Guinea form a clade, sister to *P*. *pulvinata* sp. nov. All together, they consitute the *Parahesione* clade which, in turn, is sister to the *Amphiduros*–*Amphiduropsis* clade (Fig. 10). K2P genetic distance between the two new species is 11.0% (10.1% uncorrected).

## Discussion

*Parahesione luteola* was reported from oyster shell and burrows of *Upogebia affinis* (Say, 1818) in the Atlantic coast of the United States of America (Pettibone, 1963) and is regarded as a facultative symbiont (Martin & Britayev, 1998). Unidentified species of *Parahesione* were reported from the Arctic Sea (Atkinson & Percy, 1991), Australia (Gunton et al., 2021), Costa Rica (Maurer, Vargas & Dean, 1988), New Caledonia (Ruta et al., 2007) and Vietnam (Britayev & Antokhina, 2012). The Vietnamese specimen is here considered as belonging to *P. pulvinata* sp. nov. as suggested by Britayev & Antokhina (2012) and discussed above in the corresponding *Remarks* section. The specimen of *Parahesione* sp. collected by Ruta et al. (2007) in New Caledonia (Pacific Ocean), is far from the type locality of the single known species of the genus, the eastern Atlantic coast of the United States (Pettibone, 1956). We reexamined the specimen used in Ruta et al. (2007) and confirmed that the specimen has six enlarged cirri and dorsal cirri without dorsal foliose lobe (Figs. S2A and S2B) and is likely another new species but is not described here. Additionally, we examined the specimens of *P*. *luteola* in the USNM (Figs. S2C and S2D) and they have six enlarged cirri and dorsal cirri without dorsal foliose lobe agreeing with Pettibone (1956)’s description.

In this situation, we assign these two new species to the same genus *Parahesione* and modify the diagnosis of the genus. However, the DNA repository data for *Parahesione* sp. used in Ruta et al. (2007) is very limited, with only a single partial sequence of 28 S (381 bp). We could not determine the other gene sequences because the specimen was preserved in formalin. Given the situation described above, there exists a possibility that the two morpho-types *Parahesione* (eight enlarged cirri, rectangle prostomium, dorsal cirri with dorsal foliose lobe *vs*. six enlarged cirri, trapezoidal prostomium, dorsal cirri without dorsal foliose lobe) could be distinguished on a molecular phylogenetic tree if they are re-examined with additional specimens and gene sequences. To validate whether these characteristics are indicative of phylogenetic affiliations, the necessity for further additional sampling is unequivocal.

Our phylogenetic results (Fig. 10) showed that *Parahesione* as closest to the *Amphiduros*-*Amphiduropsis* clade consistent with Ruta et al. (2007), and supporting its inclusion in Amphidurini as suggested by Pleijel et al. (2012). *Amphiduros* and *Amphiduropsis* also have enlarged dorsal cirri on segments 1–5, but bear a short median antenna, distinguishing them from *Parahesione*.

Despite their obvious morphological and molecular differences, *Parahesione apiculata* sp. nov. and *P. pulvinata* have always been found inside ghost shrimp burrows, suggesting they are obligate symbionts. Moreover, they have always been found in association with *G*. *armatus* and with *N. jousseaumei* and *Upogebia* sp. (the latter still requiring a more precise identification, N. Jimi, 2022, personal observation), thus suggesting a high degree of host specificity. Moreover, like many other symbiotic polychaetes (Martin & Britayev, 1998, 2018), both species show morphological adaptations to symbiosis. These include flat bodies and dorsal foliose lobes, which is not found in the non-symbiotic species of the *Amphiduropsis*-*Amphiduros* sister clade. Flat bodies have been reported for symbiotic polynoids living in association with tube dwelling chaetopterids, which also have to move between the host body and the tube walls (Britayev et al., 2017; Britayev & Martin, 2021). Another interesting adaptation is the extreme flatness of body and dorsal foliose lobe. We suggest these features may facilitate the worm movement between the host body and the walls of the narrow burrowsand to increase the body surface either to be in contact with the host or with the burrow walls. Body expansions in symbiotic polychaetes have been only previously reported for *Gastrolepidia clavigera* Schamarda, 1861, which shows ventral sucker-like lobes increasing the body surface in contact with the slippery holothurian host body and, combined with body arching, probably have a sucker-like function (Gibbs, 1971; Britayev & Zamyshliak, 1996). The other possible function of the the dorsal foliose lobes may also be related to the efficiency of gas exchange under conditions of hypoxia in host burrows.

The bodies of the two new species also have bright red-colour when alive. Again, this contrasts with the free-living species of *Amphiduropsis*-*Amphiduros* clade, suggesting this trait was newly acquired in *Parahesione*. A bright red color was also reported for *Hesperonoe* (Polynoidae), which also live in association with mud shrimps (Sato et al., 2001; Hong, Lee & Sato, 2017), while some crustacean-associated mollusks have red blood cells that are considered as an adaptation to thrive in the burrow hypoxic conditions (Goto et al., 2018). Therefore, we agree with Martin & Britayev (2018), who suggested that red bodies (likely associated to the presence of dissolved pigment) in *Hesperonoe* may be an adaptation to live in the burrows’ hypoxic environment. Thus, further anatomical and histological studies are needed to confirm the gas exchange function of the foliose lobes and the presence of red bodies and thus assess whether they are adaptations of *Parahesione* to life under hypoxic conditions.

## Conclusions

The genus *Parahesione*, belonging to Hesionidae, is a rare group of symbiotic polychaetes living in ghost shrimp burrows with two different sets of tentacular cirri; in one species there are only six whereas in the other there are eight. Interestingly, the prostomium have different shape being trapezoidal in those species with six pairs of tentacular cirri, and rectangular for those having eight pairs of tentacular cirri. Further, the dorsal parapodial modifications involving a foliose dorsal projection has been only reported in those species with eight pairs of tentacular cirri, whereas it has not been recorded in the only species having six pairs of tentacular cirri. We have discovered two new *Parahesione* species associated with ghost shrimps from the northwest Pacific. Both species are characterized by a flattened body, expanded foliose bases of cirrophores, and a bright red color. We consider these features as adaptations to thrive in the burrow hypoxic conditions. Reconstruction of the phylogenetic tree using four genes revealed their close relationship with non-symbiotic species of the sister clade *Amphiduropsis*-*Amphiduros*, suggested the independent establishment of symbiosis in various clades of the family Hesionidae.

## Supplemental Information

10.7717/peerj.16346/supp-1 Supplemental Information 1 *Parahesione pulvinata* sp. nov., live specimen (SIO-BIC A13742, Papua New Guinea). A, anterior end, dorsal view; B, enlarged view of anterior end, dorsal view; C, middle segments, dorsal view; D, posterior segments, dorsal view. Scale bars: A–B, 5 mm; C–D, 2 mm; E–F, 1 mm.

10.7717/peerj.16346/supp-2 Supplemental Information 2 *Parahesione* sp. (A–B) used in Ruta et al. (2007) and *Parahesione luteola* (C–D) used in Pettibone (1956). A, anterior end, dorsal view; B, anterior end, ventral view; C, anterior end, lateral view; D, anterior end, ventral view.

10.7717/peerj.16346/supp-3 Supplemental Information 3 Gene sequences.

## Additional Information and Declarations

### DNA Deposition

The following information was supplied regarding the deposition of DNA sequences: The sequences of Parahesione apiculata and *P. pulvinata* are available at GenBank: OP404166, OP404167, OP407536, OP407537, OP407566, OP407585, and OP407586.

### Data Availability

The following information was supplied regarding data availability: The gene sequences are available at GenBank: OP404166, OP404167, OP407536, OP407537, OP407566, OP407585, and OP407586.

### New Species Registration

The following information was supplied regarding the registration of a newly described species: Publication LSID: urn:lsid:zoobank.org:pub:6D64D9F4-0E29-4F67-B941-300E1888108C Parahesione pulvinata LSID: urn:lsid:zoobank.org:act:2E42DB94-DF8C-447A-A7F8-8C2FDA9FF4CA Parahesione apiculata LSID: urn:lsid:zoobank.org:act:1AB8DAA4-2268-445D-A3A6-9AE9C085A856
