# Supplementary figures and images for: Two new species of Parahesione (Annelida: Hesionidae) associated with ghost shrimps (Crustacea: Decapoda) and their phylogenetic relationships
Source: PeerJ. 2023 Nov 1;11:e16346. doi: 10.7717/peerj.16346 (PMC10625355; doi:10.7717/peerj.16346)

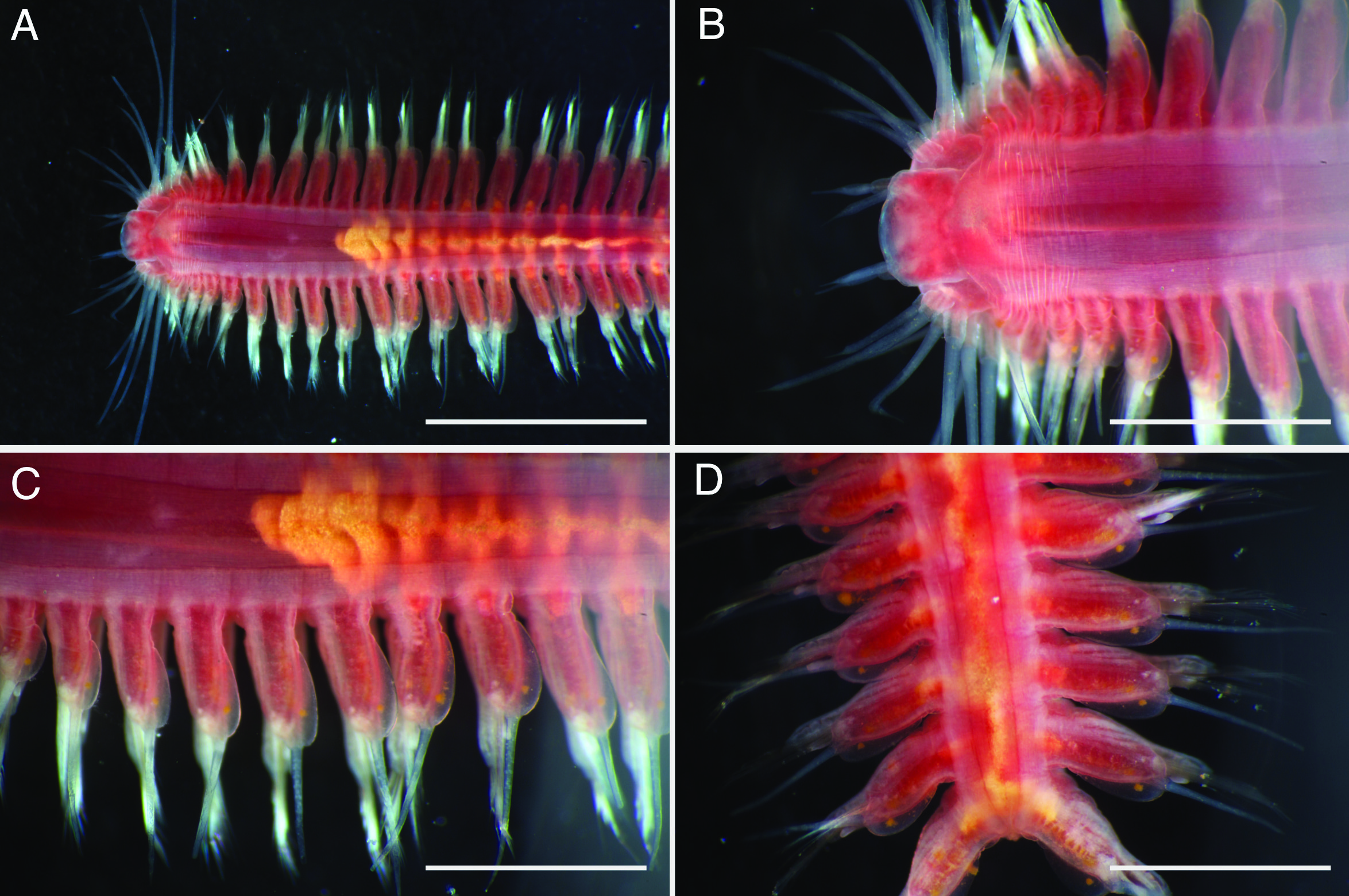

Supplement: Supplemental Information 1 — A, anterior end, dorsal view; B, enlarged view of anterior end, dorsal view; C, middle segments, dorsal view; D, posterior segments, dorsal view. Scale bars: A–B, 5 mm; C–D, 2 mm; E–F, 1 mm. [file peerj-11-16346-s001.jpg]

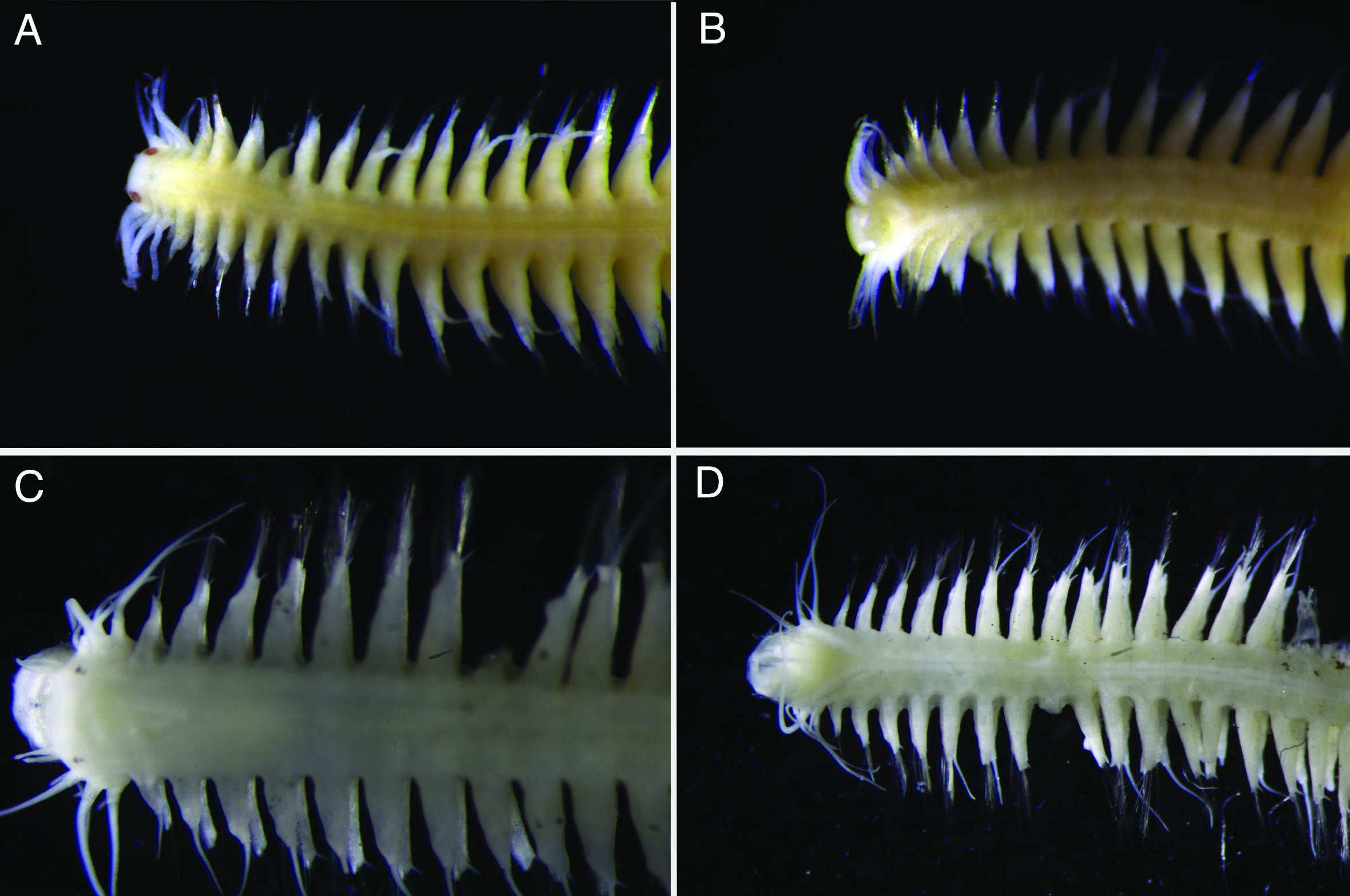

Supplement: Supplemental Information 2 — A, anterior end, dorsal view; B, anterior end, ventral view; C, anterior end, lateral view; D, anterior end, ventral view. [file peerj-11-16346-s002.jpg]
